# Quantitative and Qualitative Analysis of Flavonoids and Phenolic Acids in Snow Chrysanthemum (*Coreopsis tinctoria* Nutt.) by HPLC-DAD and UPLC-ESI-QTOF-MS

**DOI:** 10.3390/molecules21101307

**Published:** 2016-09-30

**Authors:** Yinjun Yang, Xinguang Sun, Jinjun Liu, Liping Kang, Sibao Chen, Baiping Ma, Baolin Guo

**Affiliations:** 1 Key Laboratory of Bioactive Substances and Resources Utilization of Chinese Herbal Medicine, Ministry of Education, Institute of Medicinal Plant Development, Chinese Academy of Medical Sciences & Peking Union Medical College, Beijing 100193, China; iyangyinjun@163.com (Y.Y.); liujinjun08@163.com (J.L.); sibao.chen@polyu.edu.hk (S.C.); 2 Department of Biology, Beijing Institute of Radiation Medicine, Beijing 100850, China; sxgzhwu07@163.com (X.S.); kang_liping21@163.com (L.K.); mabaiping@sina.com (B.M.)

**Keywords:** snow chrysanthemum, HPLC-DAD, UPLC-ESI-QTOF-MS, flavonoids and phenolic acids, provenance and habitat, quality evaluation

## Abstract

A simple, accurate and reliable high performance liquid chromatography coupled with photodiode array detection (HPLC-DAD) method was developed and then successfully applied for simultaneous quantitative analysis of eight compounds, including chlorogenic acid (**1**), (*R*/*S*)-flavanomarein (**2**), butin-7-*O*-β-d-glucopyranoside (**3**), isookanin (**4**), taxifolin (**5**), 5,7,3′,5′-tetrahydroxyflavanone-7-*O*-β-d-glucopyranoside (**6**), marein (**7**) and okanin (**8**), in 23 batches of snow chrysanthemum of different seed provenance and from various habitats. The results showed total contents of the eight compounds in the samples with seed provenance from Keliyang (Xinjiang, China), are higher than in samples from the other five provenances by 52.47%, 15.53%, 19.78%, 21.17% and 5.06%, respectively, which demonstrated that provenance has a great influence on the constituents in snow chrysanthemum. Meanwhile, an ultra performance liquid chromatography coupled with electrospray ionization and quadrupole time-of-flight-mass spectrometry (UPLC-ESI-QTOF-MS) was also employed to rapidly separate and identify flavonoids and phenolic acids in snow chrysanthemum from Keliyang. As a result, a total of 30 constituents, including 26 flavonoids and four phenolic acids, were identified or tentatively identified based on the exact mass information, the fragmentation characteristics, and retention times of eight reference standards. This work may provide an efficient approach to comprehensively evaluate the quality of snow chrysanthemum.

## 1. Introduction

*Coreopsis tinctoria* Nutt. (Asteraceae), native to North America, is widely cultivated around the world [1]. The capitulum of *Coreopsis tinctoria* is called “snow chrysanthemum” (Xue Ju) or “Kunlun snow chrysanthemum” (Kun Lun Xue Ju) in China [2]. The whole plant has been employed in China for hundreds of years as a folk herb for heat-clearing and detoxifying , while the capitulum of *C. tinctoria* was taken as a drink to deter diabetes in Portugal [3]. Since the beginning of this century, it has been known that snow chrysanthemum was used by the Uyghur nationality in Xinjiang (China) to prevent cardiovascular and cerebrovascular diseases, such as hypoglycemia, hypolipidemia, and hypotension. Modern pharmacological studies have not only confirmed these above effects, but also shown that it has good antioxidant activities [4,5,6]. Phytochemical studies have shown that snow chrysanthemum mainly contains flavonoids, phenolic acids, and terpenoids [7]. Flavanomarein and marein, the predominant flavonoids, were proved to have good hypoglycemic activities [3]. Meanwhile, satisfactory antioxidant activites were also reported for some of the other flavonoids, such as flavanokanin, okanin, coreopsin and flavanocorepsin [8,9,10].

The effects on cardiovascular and cerebrovascular diseases was first discovered in *C. tinctoria* from Keliyang, Xinjiang [11]. Since then, a large crop area has been dedicated to the cultivation of snow chrysanthemum in Keliyang (Xinjiang) and it is considered a Xinjiang-specific medicinal material. The good hypoglycemic and hypolipidemic effects of *C. tinctoria* have attracted wide attention in recent years [12,13,14], but the excessive planting has brought a series of problems. The influx of seeds from different sources, including inter-mating of seeds from Keliyang, as well as imported seeds, and the expansion to different areas of Xinjiang have led to unevenness of the quality of snow chrysanthemum on the market. In general, the quality of *C. tinctoria* was traditionally evaluated by the color and size of the flowers, and seeds from Keliyang were considered as superior quality, and the quality of snow chrysanthemum was better with the higher altitude.

In recent years there was some research to verify this notion. There were reports showing that the chemical composition of snow chrysanthemum was related to the ecological environment [15,16], however due to the limited samples studied and the lack of major compounds this idea was not convincing. A recently reported study about the antioxidant activities and chemical characteristics of different parts from snow chrysanthemum made the point that the contents of flavonoids and phenolic acids of snow chrysanthemum collected from Mt. Kunlun were higher than those from other regions [8,17]. To our knowledge, there is no report of relationship between seed provenance of *C. tinctoria* and its quality.

**Figure 1:**
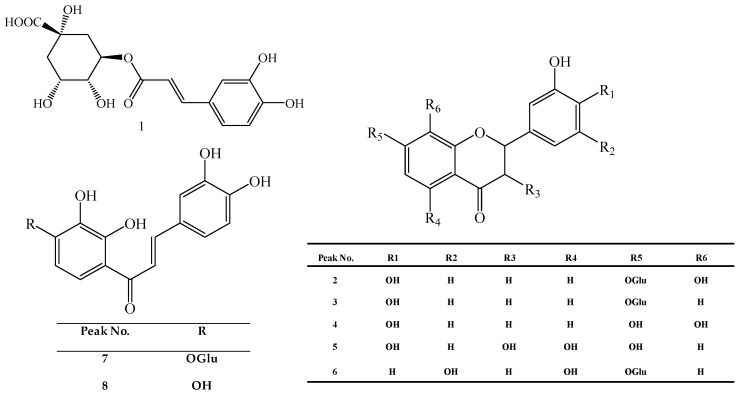
Structures of chlorogenic acid (**1**), (*R*/*S*)-flavanomarein (**2**), butin-7-*O*-β-d-glucopyranoside (**3**), isookanin (**4**), taxifolin (**5**), 5,7,3′,5′-tetrahydroxyflavanone-7-*O*-β-d-glucopyranoside (**6**), marein (**7**), and okanin (**8**).

In order to further research the effects of provenance or habitat, six batches of seeds from clearly known sources were planted at different altitudes in four regions. A consistent cultivation technique guaranteed that the differences in the quality of the snow chrysanthemums were affected only by seed provenance or habitat. A HPLC-DAD method was established to the quantification of eight compounds, namely chlorogenic acid (**1**), (*R*/*S*)-flavanomarein (**2**) [17], butin-7-*O*-β-d-gluco-pyranoside (**3**), isookanin (**4**), taxifolin (**5**), 5,7,3′,5′-tetrahydroxyflavanone-7-*O*-β-d-gluco-pyranoside (**6**), marein (**7**) and okanin (**8**) (Figure 1) in 23 batches of snow chrysanthemum grown from seeds of six different provenances in four different habitats. Meanwhile, an UPLC-ESI-QTOF-MS method was also developed to systematically identify the flavonoids and phenolic acids in snow chrysanthemum.

## 2. Result and Discussion

### 2.1. Quantitative Analysis by HPLC-DAD

#### 2.1.1. Optimization of the Extraction Method

To optimize the extraction procedure, samples (0.5 g) were extracted for 30 min using six different extraction solvents (30%, 70%, and 100% methanol and 30%, 70%, and 95% ethanol). Comparing the peak areas of the HPLC chromatograms, 30% ethanol gave poor extraction efficiency for the eight target compounds, while 70% methanol showed the best extraction effiency for all of them. Besides, ultrasonic extraction and heat reflux were also compared and the results indicated that these two methods had similar performance. Additionally, different extraction times (30, 45, and 60 min) and extraction cycles (1–3 times) were tested, too. As a result, optimal extraction condition for the active ingredients of snow chrysanthemum was determined to be one cycle of 30 mins of ultrasonic extraction with 70% methanol [18,19].

#### 2.1.2. Validation of the Method

##### Calibration Curves, Limits of Detection and Limits of Quantification

The calibration curves were generated from six different concentrations of the standard working solutions. Each concentration was analyzed in triplicate. The limits of detection (LOD) and limits of quantification (LOQ) were measured on the basis of the corresponding concentrations at signal-to-noise ratios of 3 and 10, respectively. All analytes showed good linearity of the correlation curves with correlation coefficients better that 0.9990. The LOD and LOQ values of the eight analytes varied from 3.55 to 9.25 ng and 12.46 to 35.88 ng, respectively, indicating a good sensitivity (Table 1).

##### Precision, Stability, Repeatability and Accuracy

The intra-day and inter-day precision were determined by analyzing the six replicates of the eight target standard solutions on the same day and three consecutive days, measured by calculating the relative standard deviation (RSDs) of the contents of all analytes. The RSDs of the precision of intra-day and inter-day analyses ranged from 1.76% to 4.18% and 1.63% to 4.26%, respectively (Table 2).

To test the repeatability of the method, six independently prepared samples (USA, Minfeng, Xinjiang, batch No. S1) were analyzed in parallel. Variations were expressed as RSDs of the eight target compounds, and the results were 2.51%, 3.38%, 1.77%, 2.62%, 3.74%, 2.89%, 1.58% and 1.37%, respectively. The results showed good repeatability (Table 2).

For the stability test, the same sample was analyzed at 0, 2, 4, 8, 12, and 24 h at room temperature. The RSDs of the peak areas of the eight constituents in the same sample ranged from 1.24% to 3.22%, indicating that the sample was stable under the experimental conditions (Table 2).

In order to examine the recovery of the extraction method, high amounts of the standards were added into a sample that was analyzed as described in Section 3.2.1 and Section 3.3.1. Recovery was calculated as follows:

Recovery (%) = (amount found – original amount)/spiked amount × 100%

The results showed that the mean recovery ranged from 95.75% to 103.50% with RSDs less than 4.88% for the eight compounds (Table 2).

#### 2.1.3. Quantitative Analysis of Flavonoids and Phenolic Acid in Snow Chrysanthemum by HPLC-DAD

**Figure 2:**
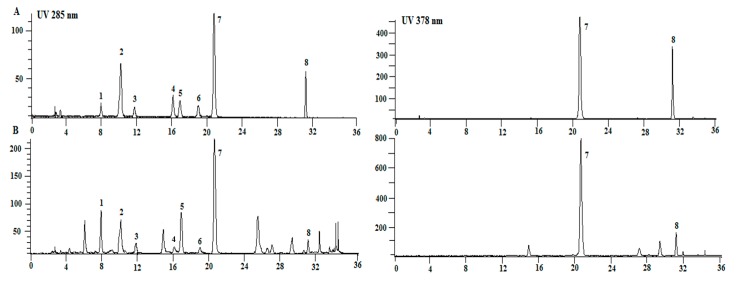
Typical HPLC chromatograms of mixed standards (**A**) and crude extract of snow chrysanthemum (**B**), detected at 285 nm (left) and 378 nm (right).

The established HPLC-DAD method was successfully applied to the determination of chlorogenic acid (**1**), (*R*/*S*)-flavanomarein (**2**), butin-7-*O*-β-d-glucopyranoside (**3**), isookanin (**4**), taxifolin (**5**), 5,7,3′,5′-tetrahydroxyflavanone-7-*O*-β-d-glucopyranoside (**6**), marein (**7**) and okanin (**8**) in 23 batches of snow chrysanthemum. Figure 2 shows typivcal chromatrograms.

Due to the different seed provenance and habitat, there were remarkable differences between the samples in terms of the total contents of the investigated compounds which ranged from 73.99 mg/g–136.38 mg/g, as shown in Table 3. In addition, the contents of four flavone glycosides, including (*R*/*S*)-flavanomarein (**2**), butin-7-*O*-β-d-glucopyranoside (**3**), 5,7,3′,5′-tetrahydroxy-flavanone-7-*O*-β-d-glucopyranoside (**6**), and marein (**7**), accounted 74.46%–83.69% of the eight investigated compounds in all tested samples.

Among the four flavone glycosides, flavanomarein and marein, which both exhibited various good biological activities in vitro, accounted for a large percentage of the flavonoids (Table 3). As shown in Table 3, through the 23 batches of samples, the content of flavanomarein or marein was mostly highest in all samples with a seed source from Keliyang, and the the contents of both compounds represented more than 52% of the investigated compounds. Thus, flavanomarein and marein could plays a key role in the quality assessment of snow chrysanthemum.

**Figure 3:**
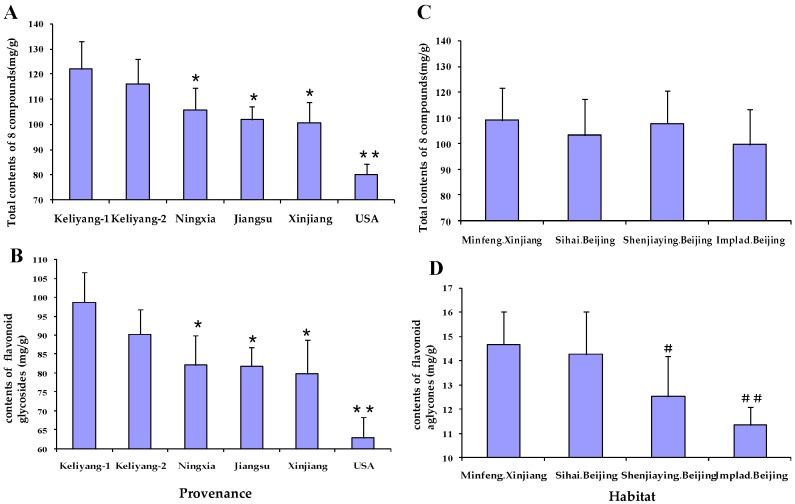
Content determination of eight compounds in snow chrysanthemum of six batches of different provenance (**A**); flavonoid glycosides (**2** + **3** + **6** + **7**) in snow chrysanthemum of six batches of different provenance (**B**); eight compounds in snow chrysanthemums from four different habitats (**C**); and flavonoid aglycones (**4** + **5** + **8**) (**D**) in snow chrysanthemums from four different habitats. Values are mean ± S.D. ** *p* < 0.01, * *p* < 0.05 vs. Keliyang-1; ^##^ *p* < 0.01, ^#^ *p* < 0.05 vs. Minfeng Xinjiang.

As shown in Figure 3A, there was significant difference in the total contents of the eight compounds between the six snow chrysanthemum seed sources. According to the folk tradition, snow chrysanthemum with seed provenance from Keliyang is considered to be the genuine herbal medicine, which is supported by the fact that the total contents of the eight compounds in snow chrysanthemum with Keliyang provenance seed were much higher than that of the four other seed source samples (*p* < 0.05). There was no difference in the total contents of the eight compounds between Keliyang-1 and Keliyang-2 provenance. Meanwhile, the contents of eight compounds in three other seed sources of snow chrysanthemum (seeds from Ningxia, Jiangsu, and Xinjiang) also showed no difference. As the major contributors of the components, the contents of flavone glycosides followed the same rule as the total components (Figure 3B).

On the other hand, the total contents of the eight compounds in snow chrysanthemum showed no significant regularity in planting in the four different habitats. The total contents of eight compounds, flavonoid glycosides and two major flavonoid glycosides in samples from the habitat of Minfeng (Xinjiang) with higher altitude were not higher than those from other three habitats. Also the total contents of the eight compounds in samples from the habitat of IMPLAD (Beijing) with lower altitude were also not lower than those from three other higher altitude habitats (Figure 3C). Therefore, the idea that the higher the attitude, the better the quality remains to be confirmed. What is interesting, however, is that in Figure 3D, the total contents of flavones aglycones, including isookanin (**4**), taxifolin (**5**), and okanin (**8**) in snow chrysanthemum planted in Minfeng (Xinjiang) were typically higher than that in samples from IMPLAD (Beijing, China) (*p* < 0.01). That may be caused by the different habitats, but there were less differences between the samples planted in Minfeng, Xinjiang and Sihai, Beijing (*p* > 0.05).

Besides, there were two batches of seeds from inter-mating from seeds of Keliyang. One was amphichrome, and the other had red flowers. In most snow chrysanthemums, the outside of the ligulate flower is yellow, and the center is red, so the flowers are basically amphichrome. The phenomomenon that the whole of flower is red is also seen in a few individuals.

**Figure 4:**
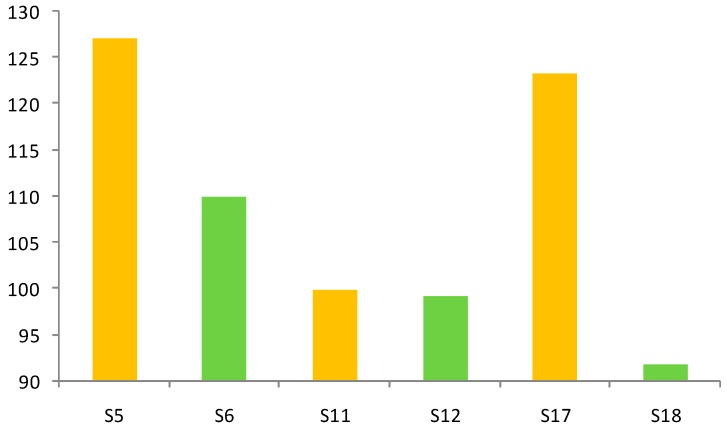
Content determination of seven flavonoids in samples which seeds were from Keliyang (Xinjiang).

For consumption as a tea, the completely red flowers are considered the most desirable, not the amphichrome ones. In the study, the red flower samples were selected from the autologous breeding of the same batch of amphichrome plants. The similar genetic background made them comparable. According to report, the red parts of flower are due to the presence of anthocyanins which have the same upstream metabolic pathways as flavonoids [20]. As shown in Figure 4, the contents of seven flavonoids in amphichrome samples (S5, S11, and S17) were comparatively higher than those in red flower ones (S6, S12, and S18) in this study. This may be explained by the fact that more anthocyanins were synthesized in red flowers and less flavonoids were synthesized in the biosynthetic pathway.

### 2.2. Identification of Flavonoids and Phenolic Acid in Snow Chrysanthemum by UPLC-MS

**Figure 5:**
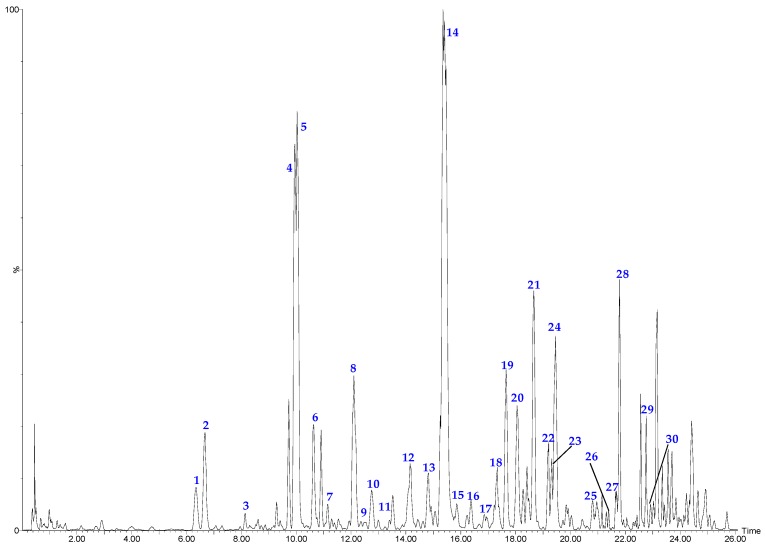
The base peak chromatograms of snow chrysanthemum by UPLC-ESI-QTOF-MS in negative ion mode.

Optimized chromatographic conditions, including mobile phase, elution program and spectrometer conditions were determned through trials in this study. As a result, a linear gradient elution with acetonitrile and water containing formic acid as the mobile phase gave the best peak resolution. The parameters of flow rate of gas, gas pressure, spray voltage, capillary temperature and voltage of entrance were optimized to obtain appropriate ionization. Figure 5 presents a typical chromatogram of a sample with mass spectrometric detection in negative ion mode using the optimum condition. A total of 30 compounds (Figure 5 and Figure 7), including four phenolic acids, 11 flavanones, six chalcones, two flavones, four flavonols and three aurones were identified in the snow chrysanthemum samples. Among them, eight compounds were unambiguously identified by comparing their retention time, the accurate masses and fragment ions with those of reference compounds and 22 compounds were tentatively assigned by matching the empirical molecular formula with that of the known compounds previously reported in the literature (Table 4).

#### 2.2.1. Identification of Phenolic Acids in Snow Chrysanthemum by ESI-Q-TOF-MS

**Figure 6:**
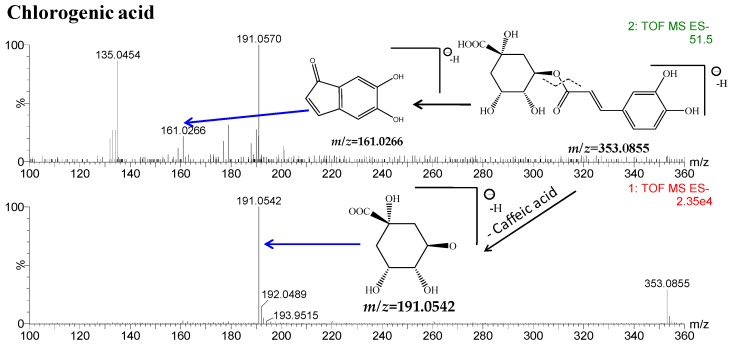

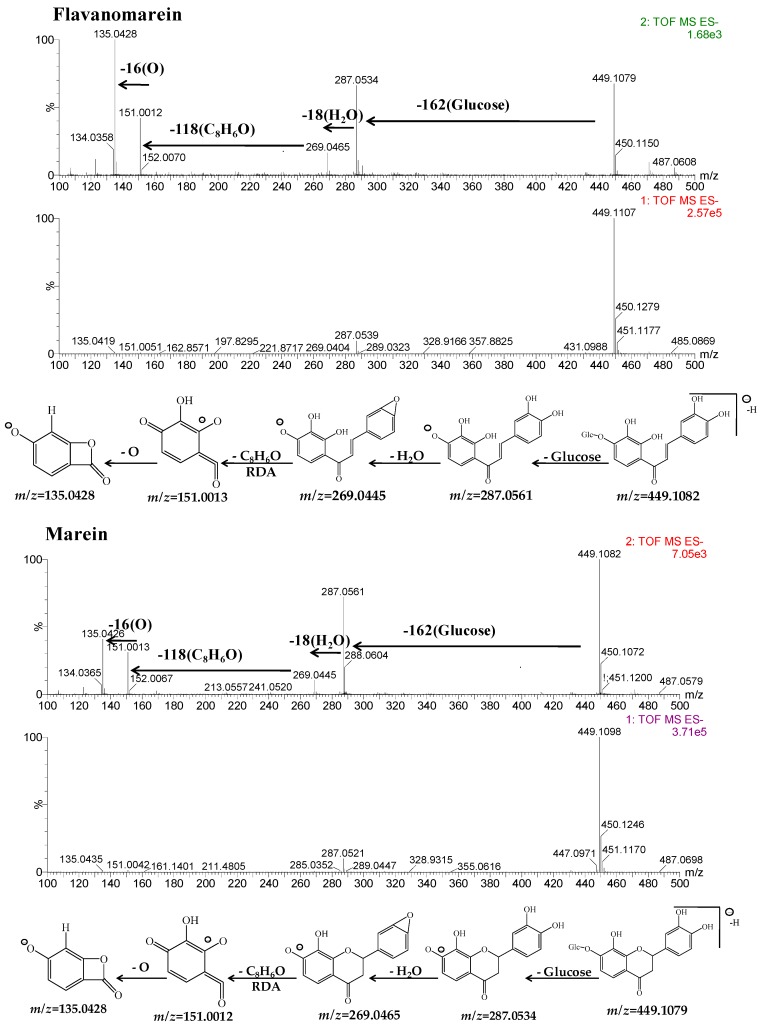
MS spectra and the proposed fragmentation pathways of chlorogenic acid, flavanomarein and marein.

Chlorogenic acid (peak 1, 6.34 min) showed a major molecular ion at *m/z* 353.0855 [M − H]^−^ (calculated for C_16_H_17_O_9_, 353.0873). At high CE, two characteristic fragment ions at *m/z* 191.0542 and 161.0266 were observed due to the cleavage of the caffeoyl bond, as shown in Figure 6. Likewise, peaks 18, 19 and 22 all gave [M − H]^−^ ions at *m*/*z* 515.1188, corresponding to C_25_H_24_O_12_. These peak produced two successive neutral losses of caffeic acid, yielding two stable fragment ion at *m/z* 353.0845 [M − H − C_9_H_6_O_3_]^−^, 191.0536 [M − H − 2C_9_H_6_O_3_]^−^. According to their empirical molecular formulae and literature data [21], peaks 18, 19 and 22 belonged to caffeoyl quinic acids and were tentatively identified as 1,3-dicaffeoylquinic acid, 3,5-dicaffeoylquinic acid, 3,4-dicaffeoylquinic acid, respectively.

#### 2.2.2. Identification of Flavonoids in Snow Chrysanthemum by ESI-Q-TOF-MS

##### The Fragmentation Rules of Standards

The ion-fragmentation pathways of flavonoids often show a retro-Diels-Alder rearrangement in ring C, and the loss of neutral molecules of H_2_O, sugar and carbonyl groups. Among the seven flavonoid standards, there were two major aglycones, flavanones (flavanomarein, butin-7-*O*-β-d-glucopyranoside, isookanin, taxifolin, 5,7,3′,5′-tetrahydroxyflavanone-7-*O*-β-d-glucopyranoside) and chalcones (marein, okanin). The two aglycones have hydroxyls at C-3′, 4′, or 5′ in the B ring and the loss of H_2_O caused by the 3′,4′-dihydroxyl disposition. In addition, these two aglycones have a hydroxyl and a sugar at C-5 and C-7 in the A ring, usually identified as being glucose.

Flavanomarein (peak 5, 10.03 min) showed a predominant molecular ion at *m/z* 449.1079 [M − H]^−^ in negative ion mode. Other fragment ions at *m/z* 287.0534 [M − H − glucose]^−^, 269.0465 [M − H − glucose − H_2_O]^−^, 151.0012 [M − H − glucose − H_2_O − C_8_H_6_O]^−^, 135.0428 [M − H − glucose − H_2_O − C_8_H_6_O − O]^−^ were assigned to the loss of H_2_O or loss of sugar, as well as the retro-Diels-Alder rearrangement, as proposed in Figure 6. The sugar at the 7 position was identified as glucose and the loss of water arose from the 3′,4′-dihydroxy substitution. The loss of C_8_H_6_O suggested that no hydroxyl was at C-3 in ring C. Similarly, the [M − H]^−^ fragmentation pathway of butin-7-*O*-β-d-glucopyranoside (peak 6, 10.63 min) at *m/z* 433.1136 indicated no hydroxyls at the 5, 6 or 8 position in ring A. Isookanin (peak 9), as the aglycone of flavanomarein, exhibited the same fragment ions as flavanomarein. Likewise, taxifolin (peak 11) eluted at 12.74 min, exhibiting [M − H]^−^ ions at *m/z* 303.0492. The loss of sugar, 3′,4′-dihydroxyl and 3-hydroxyl caused the fragment ions at *m/z* 285.0375 [M − H − H_2_O]^−^, 151.0034 [M − H − H_2_O − C_8_H_6_O_2_]^−^. The loss of C_8_H_6_O_2_ indicated the existence of a 3-hydroxyl substitution pattern. In addition, 5,7,3′,5′-tetrahydroxyflavanone-7-*O*-β-d-glucopyranoside (peak 12), another flavanone references, showed similar but different fragmentation patterns from other types of flavanones. It eluted at 14.15 min, showing four predominant fragment ions at *m/z* 449.1059 [M − H]^−^, 287.0522 [M − H − glucose]^−^, 151.0021 [M − H − glucose − C_8_H_8_O_2_]^−^. 3′,5′-dihydroxyl and retro-Diels-Alder rearrangement resulted in the loss of C_8_H_8_O_2_, without the loss of H_2_O.

Marein (peak 14, 15.40 min) produced a minor protonated ion [M − H]^−^ at *m/z* 449.1082 and a dominant fragment ion [M − H − glucose]^−^ at *m/z* 287.0561 in negative mode. The *m/z* 269.0445 [M − H − glucose − H_2_O]^−^ peak was due to the loss of water between ring the B 3′-OH and 4′-OH. In addition, a fragment ion at *m/z* 151.0013 [M − H − glucose − H_2_O − C_8_H_6_O]^−^, 135.0426 [M − H − glucose − H_2_O − C_8_H_6_O − O]^−^ was produced by a retro-Diels-Alder rearrangement. The detailed fragmentation pathway is shown in Figure 6. Okanin (peak 24) gave an [M − H]^−^ ion at *m/z* 287.0558, which was 162 Da less than marein, proving the loss of a sugar from the C-7 position compared with marein. In addition, okanin showed the similar fragmentation rules as marein.

In summary, these fragmentation behaviors of standards could be used to obtain the fragmentation rules of different types of flavonoids and the 3-OH, 5-OH or 3′,4′-dihydroxyl, 3′,5′-dihydroxyl substitution patterns could be identified by the anundance of characteristic fragments.

##### Identification of Flavanones in Snow Chrysanthemum by ESI-Q-TOF-MS

Eleven flavanones have been identified from snow chrysanthemum, six of which (peaks 4, 5, 6, 9, 11 and 12) were unambiguously identified by comparison with the reference standards. Peak 2 produced a [M − H]^−^ ion at *m/z* 465.1045 [M − H]^−^ (calculated for C_21_H_21_O_12_, 465.1033). Moderately abundant product ions at *m/z* 303.0477 and 285.0390 were formed by the neutral losses of glucose and H_2_O. The loss of H_2_O confirmed the existence of a 3′,4′-dihydroxyl substitution pattern. The fragment ions at *m/z* 151.0021 yielded by retro-Diels-Alder rearrangement in ring C and the loss of C_8_H_6_O_2_ showed the existence of a 3-OH group. By comparing its fragment ions with those of the taxifolin standard, it was identified as taxifolin-7-*O*-β-d-glucoside. Peak 7 gave an [M − H]^−^ ion at *m/z* 611.1619, corresponding to a molecular formula C_27_H_32_O_16_. At high CE, ions at *m/z* 449.1105 and 287.0395 were produced by two successive neutral losses of glucose from the deprotonated [M − H]^−^ molecule of peak 7. In addition, the fragment ion at *m/z* 269.0334 resulted from the loss of H_2_O. Likewise, the fragment ion at *m/z* 151.0042 was produced by the characteristic RDA cleavage and neutral loss of C_8_H_6_O from the fragment ion at *m/z* 269.0334. Therefore, peak 7 was tentatively identified as luteolin 7-*O*-β-d-sophoroside [22]. Similarly, peak 10 exhibited an ion [M − H]^−^ at *m/z* 433.1135 (C_21_H_22_O_10_), which produced a major fragment ion at *m/z* 271.0605 by loss of glucose, indicating the presence of a glucose unit in its structure. A fragment ion at *m/z* 135.0424 resulted from the loss of C_8_H_8_O_2_ from the fragment ion at *m/z* 271.0605. That indicated the existence of a 3′,5′-dihydroxy disposition in peak 10 without the loss of water, so peak 10 was identified as 7,3′,5′-trihydroxyflavanone-7-*O*-β-d-glucopyranoside by comparison with an authentic standard. Peak 17 gave an [M − H]^−^ at *m/z* 271.0605, corresponding to the fragment ion of peak 10. According to the fragment ions, peak 17 was identified as 7,3′,5′-trihydroxyflavanone. Similarly, by comparison with the available data, peak 25 was also identified having a 3′,5′-dihydroxyl susbtituion pattern and the major fragment ions at *m/z* 287.0534 and 151.0042 displayed the existence of a 5-OH group, so peak 25 was tentatively assigned as 5,7,3′,5′-tetrahydroxyflavanone.

##### Identification of Chalcones in Snow Chrysanthemum by ESI-Q-TOF-MS

Chalcones, one of the major type of flavonoids in the snow chrysanthemum, have similar fragment rules as flavanones, but there is a tremendous difference in chromatographic behavior between chalcones and flavanones in that chalcones have longer retention times than flavanones [18]. Peaks 14 and 24 were unambiguously identified as marien and okanin by comparison with reference standards. Peak 21 showed an obvious ion of [M − H]^−^ at *m/z* 433.1135, which was 16 Da less than that of peak 14, indicating removal of an atom of oxygen from the C-8 position of marein. Otherwise it showed similar fragment ions as marein. Thus, the structure of peak 21 was tentatively identified as coreopsin. Likewise, peak 29 displayed an ion [M − H]^−^ at *m/z* 271.0597 (C_15_H_11_O_5_). By comparison with peak 21, we found that its molecular weight was 162 Da less than that of compound 21, indicating the loss of a glucose unit from the C-7 position of peak 21. Peak 23 produced a dominant fragment ion [M − H]^−^ at *m/z* 491.1191(C_21_H_22_O_10_) in negative ion mode, which was 42 Da more than that of marein. Besides, its spectrum at high CE showed a series of ions at *m/z* 287.0522 [M − H − AcGlu]^−^, 269.0430 [M − H − AcGlu − H_2_O]^−^, 151.0021 [M − H − AcGlu − H_2_O − C_8_H_6_O]^−^, 135.0443 [M − H − AcGlu − H_2_O − C_8_H_6_O − O]^−^, indicating the existence of acetylation-glucose substitution. On the basis of the available reference data [23], peak 23 was tentatively identified as acetylmarein. Similarly, peak 26 was identified as acetylcoreopsin due to an ion at *m/z* 475.1246 [M − H]^−^ and a fragment ion at *m/z* 271.0614 produced by losses of acetylation/glucose unit (204 Da) from the deprotonated molecule ion of [M − H]^−^, which were in agreement with the data reported in the literature [24].

##### Identification of Flavones in Snow Chrysanthemum by ESI-Q-TOF-MS

In addition, based on the similar fragmentation rules of flavanones and chalcones, two flavones (peaks 3 and 20) and four flavanols (peak 8, 13, 16, and 27) were also identified. Flavones with a C-2–C-3 double bond are another type of compounds found in snow chrysanthemum. Because of the C=C in flavones, the losses of C_8_H_4_O (116 Da) or C_8_H_6_O_2_ (132 Da) were the characteristic fragment ions. Peak 3 showed a major ion at 447.0912 [M − H]^−^, which was 2 Da less than peak 4 (flavanomarein), indicating the existence of a C-2/C-3 double bond. Based on other fragment ions at *m/z* 285.0385 [M − H − glucose]^−^, 267.0663 [M − H − glucose − H_2_O]^−^, 151.0024 [M − H − glucose − H_2_O − C_8_H_4_O]^−^, 135.0417 [M − H − glucose − H_2_O − C_8_H_4_O − O]^−^, peak 3 was identified as luteoloside. Peak 20 gave a major molecule ion at 285.0396 [M − H]^−^, which was 162 Da less than that of peak 3, showing a glucose unit was removed from the C-7 position of peak 3. According to other fragment ions, peak 20 was identified as luteolin.

Peak 8 showed an ion of [M − H]^−^ at *m/z* 479.0843, corresponding to the molecular formula C_21_H_20_O_13_. At high CE, the ions at *m/z* 317.0262 [M − H − glucose]^−^, 151.0023 [M − H − glucose − C_8_H_10_O_4_]^−^, 135.0428 [M − H − glucose − C_8_H_10_O_4_ − O]^−^ were produced by the loss of glucose and RDA rearrangement in ring A. Peak 8 was tentatively identified as quercetagitin-7-*O*-glucoside on the basis of the empirical molecular formula and literature data [25]. Peak 27 gave a major molecular ion at *m*/*z* 301.0330 [M − H]^−^, which was 162 Da less than that of peak 8, showing a glucose unit was removed from the C-7 position of peak 8. Accroding to other fragment ions, peak 27 was identified as quercetin. Peak 13 produced an ion of [M − H]^−^ at *m/z* 463.0878 (C_21_H_20_O_12_) and its molecular weight was 16 Da less than that of peak 8, indicating removal of an atom of oxygen from the C-3 position of peak 8. Based on the other fragment ions at *m/z* 301.0349 [M − H − glucose]^−^, 151.0021 [M − H − glucose − H_2_O − C_8_H_6_O_3_]^−^, 135.0443 [M − H − glucose − H_2_O − C_8_H_6_O_3_ − O]^−^ and a literature report [26], peak 13 was tentatively identified as quercetin-7-*O*-β-d-glucopyranoside. Peak 16 gave the same [M − H]^−^ ion at *m/z* 463.0878 of peak 13, and other fragment ions at *m/z* 301.0349 [M − H − glucose]^−^, 151.0021 [M − H − glucose − H_2_O − C_8_H_6_O_3_]^−^, 135.0443 [M − H − glucose − H_2_O − C_8_H_6_O_3_ − O]^−^ were also consistent with those of peak 13, therefore, peak 16 was tentatively identified as hyperoside based on literature data [27].

##### Identification of Aurones in Snow Chrysanthemum by ESI-Q-TOF-MS

**Figure 7:**
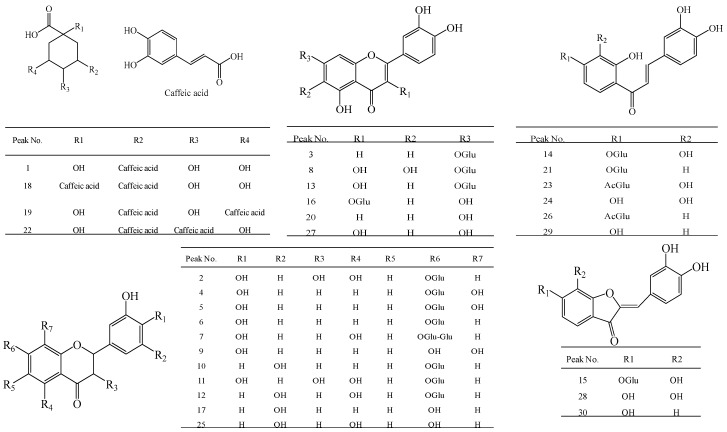
Chemical structures of the compounds identified in snow chrysanthemum.

Peak 15 produced an [M − H]^−^ ion at *m/z* 447.0928, which indicated a molecular formula of C_21_H_22_O_11_. At high CE, two characteristic fragment ions at *m/z* 285.0414 and 151.0015 were observed caused by the loss of glucose and a RDA rearrangement in ring C. Hence, peak 15 was identified as maritimein and this identification was confirmed by comparsion with the literature [28]. Likewise, peak 28 gave a major molecule ion at *m/z* 285.0385 [M − H]^−^, which was 162 Da less than that of peak 15, showing a glucose unit was removed from the C-7 position of peak 15. According to other fragment ions, peak 28 was identified as 6,7,3’,4’-tetrahydroxyaurone. Peak 30 showed an [M − H]^−^ ion at *m/z* 269.0414 and its molecular weight was 16 Da less than that of peak 28, indicating an atom of oxygen was removed from the C-8 position of peak 28. Based on other fragment ions at *m/z* 135.0357 [M − H − C_8_H_6_O_2_]^−^ and literature date [29], peak 30 was tentatively identified as sulfuretin. All the structure assigments are summarized in Figure 7.

## 3. Experimental Section

### 3.1. Samples, Standards and Reagents

Six batches of seeds were collected from the USA, Ningxia, Jiangsu, and Xinjiang. Then we planted them in four different regions: Minfeng (Xinjiang), Sihai (Beijing), Shenjiaying (Beijing), and Implad in Beijing. Detailed information about the seeds and regions is shown in Table 5.

Chlorogenic acid (≥98%) was purchased from Muenster (Chengdu, China). Flavanomarein, marein, isookanin, okanin, 5,7,3′,5′-tetrahydroxyflavanone-7-*O*-β-d-glucopyranoside, taxifolin, and butin-7-*O*-β-d-glucopyranoside (≥98%) were separated and purified in our laboratory. Their structure were also confirmed based on their MS and NMR data [30] (Figure 1). Acetonitrile (HPLC grade) was purchased from Fisher Scientific Co. (Loughborough, UK). Formic acid (HPLC grade) was purchased from Acros Co. Ltd. (Fair Lawn, NJ, USA). Water was purified with a Mill Q-Plus system (Millipore, Billerica, MA, USA). Other reagents used for extraction and separation were AR grade, and purchased from Beijing Chemical Plant (Beijing, China).

### 3.2. Instruments and Conditions

#### 3.2.1. HPLC-DAD Analysis Conditions

HPLC-DAD analysis was performed on a Flexar system (Perkin Elmer, Waltham, MA, USA) equipped with a pump, a diode array detector (DAD), and a Totalchrom chromatographic workstation, with an Ageal Innoval ODS-2 column (4.6 mm × 250 mm, 5 µm). Gradient elution of 0.2% formic acid solution (solvent A) and acetonitrile (solvent B) at a flow rate of 1 mL/min was employed: 0–5 min, 13%–15% B; 5–12 min, 15%–18% B; 12–20 min, 18%–20% B; 20–24 min, 20%–22% B; 24–29 min, 22%–40% B. The injection volume was 20 µL, and the detection wave-length was set at 285 nm, 378 nm. Resolution factor (R) of the eight compounds with the closest peak from analysis of sample was ≥ 1.5. Number of theoretical plates (*n*) of the eight compounds was ≥11,000. Tailing factor (T) of the eight compounds was within 0.90 and 1.10. The chromatograms are shown in Figure 2.

#### 3.2.2. UPLC-ESI-QTOF-MS Analysis Conditions

UPLC-ESI-QTOF-MS analysis was carried out on an Acquity UPLC^TM^ system (Waters Corp., Milford, MA, USA) coupled with a Synapt G-1 MS system (Waters Corp., Manchester, UK). A Waters Acquity UPLC HSS T3 column (2.1 mm × 100 mm, 1.8 μm) was used for the analysis with the column temperature set at 45 °C. The mobile phase was water with 0.1% formic acid (A) and acetonitrile (B). The gradient used as follows: 0–5 min, 3%–5% B; 5–8 min, 5%–10% B; 8–15 min, 10%–14% B; 15–20 min, 14%–20% B; 20–22 min, 20%–30% B; 22–28 min, 30%–35% B .The flow of rate was 0.5 mL/min, and the injection volume was 2 µL. The TOF MS detection was performed on a Synapt MS system with the data acquisition modern MS^E^. The tandem mass experiment was performed in negative ESI ionization modes with the data acquisition ranging from 100 to 1500 Da. The source temperature was 100 °C, and the desolvation temperature was 450 °C with desolvation gas flow of 900 L/h. Leucine Enkephalin (200 pg/μL) was used as the lock mass. The capillary voltage was 2.5 KV, and the cone voltage was 40 V. The collision energy was 6 eV (trap) and 4 eV (transfer) for low-energy scans, and 45–60 eV ramp (trap) and 12 eV (transfer) for high-energy scans. Finally, the instrument was controlled by Waters Masslynx 4.1 software.

### 3.3. Sample and Standard Solution Preparation for Analysis

#### 3.3.1. Sample Preparation

Sample (about 0.5 g) were accurately weighed into a covered 50 mL conical flask. Then, 70% methanol (20 mL) was accurately added into the flask. The mixture was ultrasonicated (500 W, 40 KHz) for 30 min, and filtered into a 25 mL volumetric flask. Afterward, the residue was washed with 70% methanol (3 mL). Finally, the sample solution was made up to 25 mL and filtered through a 0.22 µm filter for further analysis.

#### 3.3.2. Standard Solution Preparation

Each of standards were accurately weighed, dissolved in methanol and diluted with methanol to an appropriate concentration. A mixed solution of eight standards, containing 300 µg/mL of chlorogenic acid, 1000 µg/mL of flavanomarein, 1600 µg/mL of marein, 400 µg/mL of isookanin, 700 µg/mL of okanin, 350 µg/mL of 5,7,3′,5′-tetrahydroxyflavanone-7-*O*-β-d-glucopyranoside, 100 µg/mL of taxifolin, and 50 µg/mL of butin-7-*O*-β-d-glucopyranoside, was prepared in methanol and stored in the refrigerator at 4 ℃ until required for analysis.

### 3.4. Method Validation

#### 3.4.1. Calculations

Calibration curves were plotted by the peak area (Y) of analytes against concentration (X) of standards. The linear range was evaluated by linear regression analysis calculated by the least square regression method. Limits of detection (LOD) and quantification (LOQ) under the present chromatographic conditions were determined on the basis of response and slope of each regression equation at a signal-to-noise ratio (S/N) of 3 and 10, respectively.

#### 3.4.2. Precision, Repeatability, Stability and Accuracy

Intra-day and inter-day variations were chosen to determine the precision of the method. They were determined by analyzing mixed standards solution six times within one day and on three consecutive days. Then the relative standard deviation (RSD) was taken as a measure of precision. To assess the repeatability of the method, a sample was extracted and analyzed six times parallelly. Then calculate the RSDs of the contents of eight compounds. The stability test was performed by analysis of the sample at 0, 2, 4, 6, 8, 12 and 24 h, and calculating the RSDs for peak area ratio of each analyte. The recovery test was used to evaluate the accuracy of this method. Accurate amounts of eight standards were added to the known amounts of eight compounds in samples. The above-prepared samples (*n* = 6) were extracted and analyzed as described as in Section 3.2.1 and Section 3.3.1. The average recoveries were determined by the formula: Recovery (%) = (amount found − original amount)/spiked amount × 100%.

## 4. Conclusions

In this study, a high-performance liquid chromatography coupled with diode array detection (HPLC-DAD) method was established and validated in terms of linarity, sensitivity, precision, accuracy and stability, and then successfully applied for simultaneous quantitative analysis of eight characteristic compounds in snow chrysanthemum. The results of the 23 batches of samples demonstrate the influence of seed provenance and habitat. Flavanomarein and marein are found to be abundant in 23 batches of samples and could be used as suitable markers for quality control of snow chrysanthemum. In addition, a high resolution UPLC-ESI-QTOF-MS/MS method was developed for the systemic analysis of the flavonoids and phenolic acids in snow chrysanthemum. Based on their exact mass, fragmentation behaviors and retention timee, a total of 30 ingredients were identified in the crude extract of snow chrysanthemum, providing a deeper understanding of the chemical constituents of snow chrysanthemum.

## Figures and Tables

**Table 1:** Linearity of calibration curve for the eight target compounds.

Target Compounds	Regression Equation	R^2^	Linear Range (µg)	LOD (ng)	LOQ (ng)
**1**	Y = 1.308 × 10^5^X + 17129	0.9998	0.15–6.00	8.33	31.22
**2**	Y = 1.299 × 10^5^X + 45064	0.9990	0.60–24.00	6.76	16.38
**3**	Y = 1.641 × 10^5^X + 53463	0.9993	0.02–1.00	7.96	30.52
**4**	Y = 1.800 × 10^5^X − 58985	0.9995	0.20–8.00	6.66	25.86
**5**	Y = 4.328 × 10^5^X − 70904	0.9990	0.05–2.00	3.55	12.46
**6**	Y = 2.004 × 10^5^X + 13368	0.9990	0.18–7.00	5.28	21.08
**7**	Y = 8.303 × 10^5^X + 41070	0.9996	0.80–32.00	9.25	35.88
**8**	Y = 7.594 × 10^5^X + 15260	0.9993	0.35–14.00	6.88	23.58

**Table 2:** Intra-day and inter-day precisions, repeatability, stability, recovery tests of 8 target compounds.

Target Compounds	Precision (RSD, %)		Repeatability (RSD, %)	Stability	Recovery (%) (*n* = 6)	
	Intra-Day (*n* = 6)	Inter-Day (*n* = 6)		(RSD, %)	Average	RSD
**1**	2.92	2.78	2.51	2.03	95.75	4.58
**2**	3.53	4.06	3.38	3.15	99.67	4.88
**3**	2.58	1.92	1.77	3.52	99.50	2.21
**4**	2.97	1.63	2.62	1.73	96.35	2.87
**5**	4.16	3.02	3.74	3.03	98.52	2.79
**6**	3.21	2.87	2.89	2.64	98.48	2.34
**7**	1.76	3.69	1.58	1.24	102.51	3.32
**8**	4.18	4.26	1.37	2.19	103.50	1.33

**Table 3:** Content determination of eight compounds in 23 batches of snow chrysanthemum (mg·g^−1^).

NO.	1	2	3	4	5	6	7	8	Sum 2 + 7	Flavonoid Glycosides (2 + 3 + 6 + 7)	Flavonoid Aglycones (4 + 5 + 8)	Total (1 + 2 + … + 7 + 8)
S1	1.72	17.29	0.41	5.34	2.85	4.35	35.25	6.78	52.54	57.30	14.96	73.99
S2	10.09	28.59	1.09	5.07	1.83	5.84	50.02	5.66	78.61	85.54	12.56	108.19
S3	4.02	29.95	1.11	5.66	2.34	4.53	51.22	6.85	81.17	86.81	14.86	105.69
S5	5.27	26.50	1.34	6.55	2.42	12.51	70.45	7.36	96.95	110.80	16.32	132.39
S6	14.73	30.71	1.27	5.46	2.99	9.33	54.06	6.21	84.77	95.36	14.66	124.75
S7	2.08	18.61	0.40	6.11	3.19	3.33	36.86	8.92	55.47	59.20	18.22	79.51
S8	5.86	25.96	1.63	6.81	3.69	7.74	54.30	9.02	80.26	89.63	19.52	115.01
S9	5.36	24.04	0.96	4.61	1.96	4.14	49.66	7.43	73.7	78.80	14.00	98.17
S10	10.19	19.36	0.80	4.43	1.88	4.65	48.49	5.26	67.85	73.30	11.56	95.06
S11	13.93	19.76	0.66	4.44	1.35	11.01	57.24	5.43	77.00	88.67	11.22	113.83
S12	12.79	23.07	1.07	4.50	1.52	8.92	55.10	4.99	78.17	88.17	11.01	111.96
S13	2.35	18.75	0.44	3.89	2.30	2.99	45.67	7.16	64.42	67.85	13.34	83.55
S14	9.26	22.85	1.02	4.14	2.37	4.99	53.14	7.54	75.99	82.00	14.05	105.31
S15	7.58	23.05	1.27	3.53	1.87	3.70	57.36	8.72	80.41	85.37	14.12	107.07
S16	8.04	26.37	1.49	3.93	2.12	4.55	57.73	5.86	84.1	90.14	11.92	110.10
S17	12.99	29.05	1.54	3.46	1.83	15.50	65.42	6.59	94.47	111.51	11.88	136.38
S18	12.01	21.59	1.24	3.56	1.81	6.47	52.77	4.46	74.36	82.07	9.84	103.92
S19	3.75	17.45	0.58	4.28	2.60	3.96	45.26	4.89	62.71	67.25	11.77	82.78
S20	10.03	14.68	0.94	5.69	1.55	4.58	51.14	4.99	65.82	71.33	12.22	93.59
S21	8.33	16.94	1.64	4.83	1.37	6.62	51.13	5.32	68.07	76.33	11.51	96.17
S22	9.79	16.08	1.08	4.56	1.55	5.37	53.34	4.91	69.42	75.87	11.02	96.68
S23	11.11	17.89	0.75	4.55	1.22	9.90	55.34	4.27	73.23	83.88	10.04	105.04
S24	16.56	27.72	1.07	5.16	1.52	11.28	55.46	4.72	83.18	95.53	11.41	123.51

**Table 4:** Identification of 30 compounds in snow chrysanthemum.

Peak	t_R_ (min)	Formula	[M − H]^−^			Fragments Ion MS (*m*/*z*) in Negative Ion Mode	Identification
			Experimental Mass (*m*/*z*)	Theoreticalmass (*m*/*z*)	Mass Error (ppm)		
1	6.34	C_16_H_18_O_9_	353.0855	353.0873	−1.6	353.0855 [M − H]^−^ 191.0542 [M − H − C_9_H_6_O_3_]^−^	Chlorogein acid
2	6.68	C_21_H_22_O_12_	465.1045	465.1033	2.2	465.1045 [M − H]^−^ 303.0477 [M − H − glucose]^−^ 285.0390 [M − H − glucose − H_2_O]^−^ 151.0021 [M − H − glucose − H_2_O − C_8_H_6_O_2_]^−^	Taxifolin-7-*O*-β-d-glucoside
3	8.14	C_21_H_20_O_11_	447.0912	447.0927	−3.4	447.0912 [M − H]^−^ 285.0385 [M − H − glucose]^−^ 267.0663 [M − H − glucose − H_2_O]^−^ 151.0024 [M − H − glucose − H_2_O − C_8_H_4_O]^−^ 135.0417 [M − H − glucose − H_2_O − C_8_H_4_O − O]^−^	Luteoloside
4	9.95	C_21_H_22_O_11_	449.1079	449.1084	−0.7	449.1079 [M − H]^−^ 287.0534 [M − H − glucose]^−^ 269.0465 [M − H − glucose − H_2_O]^−^ 151.0012 [M − H − glucose − H_2_O − C_8_H_6_O]^−^ 135.0428 [M − H − glucose − H_2_O − C_8_H_6_O − O]^−^	*R* or *S*-flavanomarein
5	10.03	C_21_H_22_O_11_	449.1079	449.1084	−0.7	449.1079 [M − H]^−^ 287.0534 [M − H − glucose]^−^ 269.0465 [M − H − glucose − H_2_O]^−^ 151.0012 [M − H − glucose − H_2_O − C_8_H_6_O]^−^ 135.0428 [M − H − glucose − H_2_O − C_8_H_6_O − O]^−^	*R* or *S*-flavanomarein
6	10.63	C_21_H_22_O_10_	433.1136	433.1135	0.2	433.1136 [M − H]^−^ 271.0587 [M − H − glucose]^−^ 253.0482 [M − H − glucose − H_2_O]^−^ 135.0231 [M − H − glucose − H_2_O − C_8_H_6_O]^−^	Butin-7-*O*-β-d-gluco-pyranoside
7	11.13	C_27_H_32_O_16_	611.1619	611.1612	1.1	611.1619 [M − H]^−^ 449.1105 [M − H − glucose]^−^ 287.0395 [M − H − 2glucose]^−^ 269.0334 [M − H − 2glucose − H_2_O]^−^ 151.0042 [M − H − 2glucose − H_2_O − C_8_H_6_O]^−^ 135.0437 [M − H − 2glucose − H_2_O − C_8_H_6_O − O]^−^	Luteolin 7-*O*-β-d-sophoroside
8	12.10	C_21_H_20_O_13_	479.0843	479.0826	3.5	479.0843 [M − H]^−^ 317.0262 [M − H − glucose]^−^ 151.0023 [M − H − glucose − C_8_H_10_O_4_]^−^ 135.0428 [M − H − glucose − C_8_H_10_O_4_ − O]^−^	Quercetagitin-7-O-glucoside
9	12.15	C_15_H_12_O_6_	287.0545	287.0556	−1.1	287.0545 [M − H]^−^ 269.0441 [M − H − H_2_O]^−^ 151.0025 [M − H − H_2_O − C_8_H_6_O]^−^ 135.0440 [M − H − H_2_O − C_8_H_6_O − O]^−^	Isookain
10	12.35	C_21_H_22_O_10_	433.1135	433.1135	0	433.1135 [M − H]^−^ 271.0605 [M − H − glucose]^−^ 135.0424 [M − H − glucose − C_8_H_8_O_2_]^−^	7,3’,5’-Trihydroxy-flavanone-7-*O*-β-d-glucopyranoside
11	12.74	C_15_H_12_O_7_	303.0492	303.0505	−4.3	303.0481 [M − H]^−^ 285.0375 [M − H − H_2_O]^−^ 151.0034 [M − H − H_2_O − C_8_H_6_O_2_]^−^ 135.0475 [M − H − H2O − C_8_H_6_O_2_ − O]^−^	Taxifolin
12	14.15	C_21_H_22_O_11_	449.1059	449.1084	−3.2	449.1059 [M − H]^−^ 287.0522 [M − H − glucose]^−^ 151.0021 [M − H − glucose − C_8_H_8_O_2_]^−^ 135.0443 [M − H − glucose − C_8_H_8_O_2_-O]^−^	5,7,3’,5’-Tetra-hydroxyflavanone-7-*O*-β-d-gluco-pyranoside
13	14.83	C_21_H_20_O_12_	463.0878	463.0877	0.1	463.0878 [M − H]^−^ 301.0349 [M − H − glucose]^−^ 151.0021 [M − H − glucose − H_2_O − C_8_H_6_O_3_]^−^ 135.0443 [M − H − glucose − H_2_O − C_8_H_6_O_3_ − O]^−^	Quercetin-7-*O*-β-d-glucopyranoside
14	15.40	C_21_H_22_O_11_	449.1082	449.1084	−0.2	449.1082 [M − H]^−^ 287.0561 [M − H − glucose]^−^ 269.0445 [M − H − glucose − H_2_O]^−^ 151.0013 [M − H − glucose − H_2_O − C_8_H_6_O]^−^ 135.0426 [M − H − glucose − H_2_O − C_8_H_6_O − O]^−^	Marein
15	15.85	C_21_H_20_O_11_	447.0928	447.0927	0.2	447.0928 [M − H]^−^ 285.0414 [M − H − glucose]^−^ 151.0015 [M − H − glucose − C_8_H_6_O_2_]^−^ 135.0443 [M − H − glucose − C_8_H_6_O_2_ − O]^−^	Maritimein
16	16.23	C_21_H_20_O_12_	463.0878	463.0877	0.1	463.0878 [M − H]^−^ 301.0449 [M − H − glucose]^−^ 151.0021 [M − H − glucose − H_2_O − C_8_H_6_O_3_]^−^ 135.0443 [M − H − glucose − H_2_O − C_8_H_6_O_3_ − O]^−^	Hyperoside
17	16.94	C_15_H_12_O_5_	271.0605	271.0606	−0.1	271.0605 [M − H]^−^ 135.0229 [M − H − C_8_H_8_O_2_]^−^	7,3’,5’-Trihydroxy-flavanone
18	17.40	C_25_H_24_O_12_	515.1188	515.1190	−0.4	515.1188 [M − H]^−^ 353.0845 [M − H − C_9_H_6_O_3_]^−^ 191.0536 [M − H − 2C_9_H_6_O_3_]^−^	1,3-Dicaffeoylquinic acid
19	17.65	C_25_H_24_O_12_	515.1188	515.1190	−0.4	515.1188 [M − H]^−^ 353.0845 [M − H − C_9_H_6_O_3_]^−^ 191.0536 [M − H − 2C_9_H_6_O_3_]^−^	3,5-Dicaffeoylquinic acid
20	18.05	C_15_H_10_O_6_	285.0396	285.0399	−0.3	285.0396 [M − H]^−^ 267.0078 [M − H − H_2_O]^−^ 151.0080 [M − H − H_2_O − C_8_H_4_O]^−^ 135.0432 [M − H − H_2_O − C_8_H_4_O − O]^−^	Luteolin
21	18.66	C_21_H_22_O_10_	433.1135	433.1135	0	433.1135 [M − H]^−^ 271.0605 [M − H − glucose]^−^ 253.0494 [M − H − glucose − H_2_O]^−^ 135.0424 [M − H − glucose − H_2_O − C_8_H_6_O]^−^	Coreopsin
22	19.20	C_25_H_24_O_12_	515.1188	515.1190	−0.4	515.1188 [M − H]^−^ 353.0845 [M − H − C_9_H_6_O_3_]^−^ 191.0536 [M − H − 2C_9_H_6_O_3_]^−^	3,4-Dicaffeoylquinic acid
23	19.32	C_23_H_24_O_12_	491.1191	491.1190	0.2	491.1191 [M − H]^−^ 287.0522 [M − H − AcGlu]^−^ 269.0430 [M − H − AcGlu − H_2_O]^−^ 151.0021 [M − H − AcGlu − H_2_O − C_8_H_6_O]^−^ 135.0443 [M − H − AcGlu − H_2_O − C_8_H_6_O − O]^−^	Acetylmarein
24	19.45	C_15_H_12_O_6_	287.0558	287.0556	0.7	287.0558 [M − H]^−^ 269.0430 [M − H − H_2_O]^−^ 151.0021 [M − H − H_2_O − C_8_H_6_O]^−^ 135.0443 [M − H − H_2_O − C_8_H_6_O − O]^−^	Okanin
25	20.79	C_15_H_12_O_6_	287.0534	287.0556	−2.2	287.0534 [M − H]^−^ 151.0042 [M − H − C_8_H_8_O_2_]^−^ 135.0426 [M − H − C_8_H_8_O_2_ − O]^−^	5,7,3’,5’-Tetrahydroxy-flavanone
26	21.40	C_23_H_24_O_11_	475.1246	475.1240	0.6	475.1246 [M − H]^−^ 271.0614 [M − H − AcGlu]^−^ 253.0498 [M − H − AcGlu − H_2_O]^−^ 135.0424 [M − H − AcGlu –H_2_O − C_8_H_6_O]^−^	Acetylcoreopsin
27	21.67	C_15_H_10_O_7_	301.0330	301.0348	−1.8	301.0330 [M − H]^−^ 285.0385 [M − H − H_2_O]^−^ 151.0021 [M − H − H_2_O − C_8_H_6_O_2_]^−^	Quercetin
28	21.79	C_15_H_10_O_6_	285.0385	285.0399	−1.3	285.0385 [M − H − ]^−^ 151.0025 [M − H − C_8_H_6_O_2_]^−^ 135.0428 [M − H − C_8_H_6_O_2_ − O]^−^	6,7,3’,4’-Tetrahydroxy-aurone
29	22.76	C_15_H_12_O_5_	271.0597	271.0606	−0.9	271.0597 [M − H]^−^ 253.0496 [M − H − H_2_O]^−^ 135.0428 [M − H − H_2_O − C_8_H_6_O]^−^	Butein
30	23.03	C_15_H_10_O_5_	269.0414	269.0450	−3.6	269.0414 [M − H]^−^ 135.0357 [M − H − C_8_H_6_O_2_]^−^	Sulfuretin

**Table 5:** Summary of the analyzed samples.

Sample Codes	Provenance	Habitat
S1	USA, purchased from Jiangsu Pacific Seeds, Chenmei.	Minfeng (Xinjiang, H = 2700 m)
S2	Ningxia, purchased from Jiangsu Pacific Seeds, Chenmei.	
S3	Jiangsu, purchased from Jiangsu Pacific Seeds, Chenmei.	
S4 ^※^	Xinjiang, purchased from Xinjiang *Coreopsis tinctoria* Company	
S5	Xinjiang, intermating from seeds of Keliyang, amphichrome, purchased from Xinjiang *Coreopsis tinctoria* Company.	
S6	Xinjiang, intermating from seeds of Keliyang, red flowers, purchased from Xinjiang *Coreopsis tinctoria* Company.	
S7	USA, purchased from Jiangsu Pacific Seeds, Chenmei.	Sihai (Beijing, H = 725 m)
S8	Ningxia, purchased from Jiangsu Pacific Seeds, Chenmei.	
S9	Jiangsu, purchased from Jiangsu Pacific Seeds, Chenmei.	
S10	Xinjiang, purchased from Xinjiang *Coreopsis tinctoria* Company.	
S11	Xinjiang, intermating from seeds of Keliyang, amphichrome, purchased from Xinjiang *Coreopsis tinctoria* Company.	
S12	Xinjiang, intermating from seeds of Keliyang, red flower, purchased from Xinjiang *Coreopsis tinctoria* Jiangsu Pacific Seeds, Chenmei.	
S13	USA, purchased from Jiangsu Pacific Seeds, Chenmei.	Shen Jiaying (Beijing, H = 514 m)
S14	Ningxia, purchased from Jiangsu Pacific Seeds, Chenmei.	
S15	Jiangsu, purchased from Jiangsu Pacific Seeds, Chenmei.	
S16	Xinjiang, purchased from Xinjiang *Coreopsis tinctoria* Company	
S17	Xinjiang, intermating from seeds of Keliyang, amphichrome, purchased from Xinjiang *Coreopsis tinctoria*c Company.	
S18	Xinjiang, intermating from seeds of Keliyang, red flower, purchased from Xinjiang *Coreopsis tinctoria* Company.	
S19	USA, purchased from Jiangsu Pacific Seeds, Chenmei.	IMPLAD (Beijing, H = 50 m)
S20	Ningxia, purchased from Jiangsu Pacific Seeds, Chenmei.	
S21	Jiangsu, purchased from Jiangsu Pacific Seeds, Chenmei.	
S22	Xinjiang, purchased from Xinjiang *Coreopsis tinctoria* company.	
S23	Xinjiang, intermating from seeds of Keliyang, amphichrome, purchased from Xinjiang *Coreopsis tinctoria* Company.	
S24	Xinjiang, intermating from seeds of Keliyang, red flower, purchased from Xinjiang *Coreopsis tinctoria* Company.	

※ Sample S4 was lost.
